# Urinary peptide panel for prognostic assessment of bladder cancer relapse

**DOI:** 10.1038/s41598-019-44129-y

**Published:** 2019-05-21

**Authors:** Magdalena Krochmal, Kim E. M. van Kessel, Ellen C. Zwarthoff, Iwona Belczacka, Martin Pejchinovski, Antonia Vlahou, Harald Mischak, Maria Frantzi

**Affiliations:** 1 Mosaiques Diagnostics GmbH, Hannover, Germany; 2 Department of Pathology, Erasmus MC Cancer Institute, Erasmus Medical Center, Rotterdam, The Netherlands; 3 Department of Urology, Erasmus MC Cancer Institute, Erasmus Medical Center, Rotterdam, The Netherlands; 4 Biotechnology Division, Biomedical Research Foundation, Academy of Athens (BRFAA), Athens, Greece

**Keywords:** Prognostic markers, Molecular medicine, Bladder

## Abstract

Non-invasive tools stratifying bladder cancer (BC) patients according to the risk of relapse are urgently needed to guide clinical intervention. As a follow-up to the previously published study on CE-MS-based urinary biomarkers for BC detection and recurrence monitoring, we expanded the investigation towards BC patients with longitudinal data. Profiling datasets of BC patients with follow-up information regarding the relapse status were investigated. The peptidomics dataset (n = 98) was split into training and test set. Cox regression was utilized for feature selection in the training set. Investigation of the entire training set at the single peptide level revealed 36 peptides being strong independent prognostic markers of disease relapse. Those features were further integrated into a Random Forest-based model evaluating the risk of relapse for BC patients. Performance of the model was assessed in the test cohort, showing high significance in BC relapse prognosis [HR = 5.76, p-value = 0.0001, c-index = 0.64]. Urinary peptide profiles integrated into a prognostic model allow for quantitative risk assessment of BC relapse highlighting the need for its incorporation in prospective studies to establish its value in the clinical management of BC.

## Introduction

Bladder cancer (BC) is among the most common and costliest malignancies worldwide^1^. Although the majority of BC cases are non-muscle invasive (NMIBC), BC is characterized by high recurrence (~70%) and progression rates (10–20%) to muscle-invasive disease (MIBC)^2–4^. As such, NMIBC patients undergo life-long surveillance through invasive cystoscopy. Based on the guidelines, different treatment schemes are recommended for NMIBC^5^ and MIBC^6^. High-grade NMIBC patients are treated with Bacillus Calmette-Guérin (BCG) immunotherapy or intravesical instillation of mitomycin-C/epirubicin (chemotherapy)^5^, while MIBC patients undergo radical cystectomy^6^. Extensive genomic characterization of BC revealed high tumor heterogeneity indicating the existence of distinct disease molecular subtypes^7,8^. In fact, growing evidence suggests that BC represents a group of heterogeneous diseases, both molecularly and clinicopathologically^9,10^.

As novel therapeutic interventions for BC are on the rise, including immune checkpoint inhibitors targeting Programmed cell Death (PD)-1 receptor and its ligand PD-L1, as well as cytotoxic T-lymphocyte-associated Protein 4 (CTLA4), guiding intervention through the stratification of BC patients according to the risk for relapse and/or to the predicted drug response becomes even more critical in the selection of optimal treatment approach. Therefore, complementary biomarkers are still needed to improve prognostic certainty and guide clinical intervention.

So far, a risk assessment approach is applied for identifying probabilities of recurrence and progression. Currently used risk calculators are based on clinical and pathological characteristics. Major predictive models that are used in clinical practice include^11^: (1) the European Organization for Research and Treatment of Cancer (EORTC) risk tables for the probabilities of recurrence and progression after TURBT^12^; (2) the Spanish Urological Club for Oncological Treatment scoring model (CUETO) for the risk of recurrence and progression after BCG therapy^13^; and (3) the updated EORTC risk groups for recurrence, progression, and disease-specific and overall survival for high-risk NMIBC patients receiving the BCG maintenance therapy^14^. The initial EORTC risk tables were constructed based on the six most relevant predictors of outcomes i.e. tumor stage and grade, number and size of tumors, carcinoma *in situ* (CIS), and prior recurrence rate. To account for the patients treated with BCG (low in number during EORTC risk tables’ development), an optimized scoring model was developed by CUETO introducing 1062 BCG-treated patients and including age and gender in the risk assessment equation. Updated EORTC risk tables for early recurrence include: prior recurrence rate, number of tumors and grade as main parameters, while variables in the late recurrence model consist of prior recurrence rate and number of tumors. Despite their potential in clinical practice, there are several limitations of these models, mainly involving the high complexity of the first two and over-simplicity of the latter, not being able to embrace BC heterogeneity at the molecular level. Moreover, as EORTC risk tables tend to overestimate^15^, while CUETO scoring model can underestimate the risk of disease recurrence/progression^16^, more precise prognostic models are much needed.

Urine has been already recognized as an exceptional source of biomarkers, due to the high stability of the proteome and non-invasive means of collection^17^. Moreover, urinary peptides carry substantial information not only for on-site but also for systemic events that are related to BC and depict molecular changes linked to disease pathophysiology e.g. tumor invasion and inflammation.

Mass spectrometry-derived (CE-MS) urinary profiling data have been previously explored for detection of BC^18^, as well as discrimination of non- from muscle-invasive form of BC^19^. More recently, two diagnostic panels, based on the same technology, were published for BC detection (BC-116) and monitoring of recurrence (BC-106)^20^. In the latter, the urinary profiles were also indicative of disease molecular changes during BC progression.

In this proof-of-principle study, the previously published peptidomics datasets based on the CE-MS analysis of urine from BC patients, have been evaluated in a prognostic setting for patients with available follow-up data. The aim of this investigation was dual: a) to evaluate the prognostic value of the previously published diagnostic panel (BC-106) in the form of support vector machine classifier (SVM) with regards to BC relapse and b) to investigate the prognostic value of individual peptides and apply state-of-the-art machine learning approaches for the development of a model for prognosis of BC relapse.

## Results

### Cohort characterization

Peptidomics profiles based on the urinary CE-MS analysis of 98 BC patients were evaluated according to the endpoints of BC relapse and relapse-free disease, as described in the Methods section. Out of the 98 BC patients, 45 developed a relapse during the follow-up period and 53 were relapse-free during the follow-up (Supplementary Table S1). The median follow-up time was estimated at 15.7 months (±14.6). Among the included BC patients, 78 (79%) were male and 20 (21%) were female. The detailed clinical characteristics of the study population are presented in Table 1.

**Table 1:** Patient characteristics of the study cohort (n = 98).

Age	Mean	64.4 (±11.9)
Gender	Male	78 (79%)
	Female	20 (21%)
Event	Relapse	45 (46%)
	Non-relapse	53 (54%)
Follow-up [months]	Mean	15.7 (±14.6)
Stage [previously resected tumor]	Papilloma	4
	Tis	2
	Ta	74
	T1	10
	T2	2
	T3	1
	Tx	5
Grade [previously resected tumor]	G1	22
	G2	48
	G3	17
	Gx	2
	Unknown	9
Multiplicity [previously resected tumor]	Solitary	51
	Multiple	37
	Unknown	10

No significant differences were detected with regards to age, gender, and number of events between the training and the test set.

### Association of the previously established CE-MS-based urinary diagnostic panel with BC relapse

**Figure 1:**
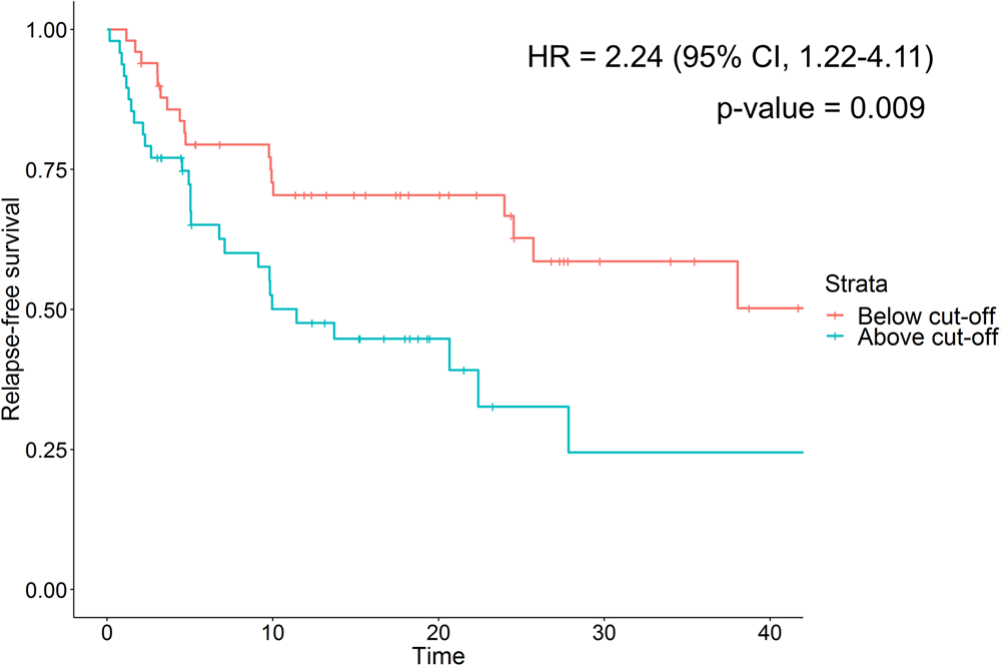
Kaplan-Mayer curve for the BC-106 score and disease-specific outcomes in the study cohorts (cut-off = −0.63 was used as reported in Frantzi *et al*.^20^) *Strata: red line – negative for recurrence*, *blue line – positive for recurrence*. *Abbreviations: HR* = *hazard ratio; CI* = *confidence interval*.

As a follow-up to the previously published study on CE-MS based biomarkers for the detection and monitoring of BC^20^, assessment of the prognostic potential of the above diagnostic biomarker panel was performed. Association of the BC-106 score^20^ with BC relapse was assessed by univariate Cox regression analysis. The results indicated a predictive value of the BC-106 diagnostic panel for disease relapse [HR = 2.24 (95% CI, 1.22–4.11), p-value = 0.009, c-index = 0.60] (Fig. 1). The CE-MS BC-106 score, measured at the baseline, was able to correctly classify 60% of the patients (n = 27), who subsequently relapsed while the cystoscopy results (at the time of sampling/ baseline) were negative (Supplementary Table S2). Moreover, the diagnostic score was significantly and inversely correlated with the time to develop the relapse (rho = −0.28, p-value = 0.005), indicating that the higher the score, the shorter the time to develop a recurrence event. These results suggested a prognostic potential of the CE-MS peptidomics profiles in prognosis of BC relapse - a hypothesis which we further explored in this study.

### Identification of BC-specific markers with prognostic potential

**Figure 2:**
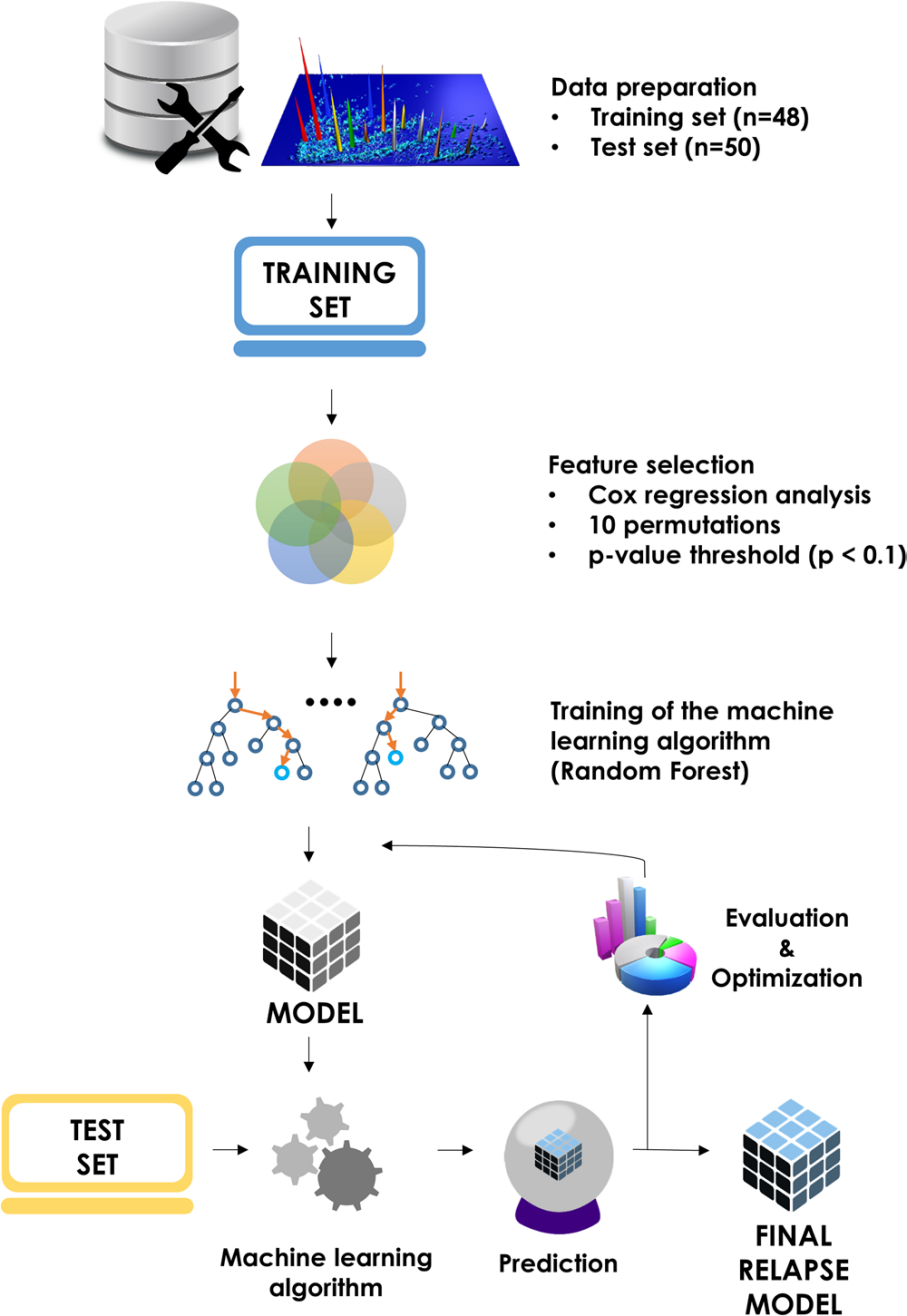
Project development workflow. The full dataset of peptidomics profiles of BC patients (n = 98) was split into training (n = 48) used for model development and test set (n = 50) retained for validation. Feature selection was performed through Cox regression analysis (10 resampling permutations) with 36 peptides found significantly predictive of the relapse (p-value < 0.1). Those were further used in the development of Random Forest-based predictive model of BC relapse. Performance of the model was evaluated on the test set and further optimized.

To fully explore the prognostic potential of the CE-MS derived profiling data, statistical analysis at the single peptide level was performed. The study workflow is presented in Fig. 2. Peptidomics profiling datasets of 98 BC patients were randomly split into a training (n = 48) and an independent test set (n = 50) assuring equal distribution of cases (relapse event) and controls (relapse-free). Median age, gender, and event distribution were not significantly different between the training and test set (Supplementary Table S1**)**. Detailed pertinent clinico-pathological information for the training and test sets are also listed in Supplementary Table S2. To assess the association of each peptide’s abundance with disease outcome and select the set of peptides (features) for machine learning model development, Cox regression analysis was performed in ten re-sampling analyses, each time by randomly discarding thirty percent of the patients. The peptide sets that were established based on the p-value threshold (p-value < 0.1) are reported in Supplementary Table S3.

### Development of machine learning model for relapse prediction

Based on the hypothesis that combination of prognostic markers into a multi-marker classifier may increase the accuracy of prediction, we tested the significant peptides, which were commonly identified as significant in six (n_sig.peptides_ = 36), seven (n_sig.peptides_ = 25), eight (n_sig.peptides_ = 16), nine (n_sig.peptides_ = 12) and ten (n_sig.peptides_ = 4) Cox regression repeated analyses as features for the development of a machine learning model (Supplementary Table S3). A random forest algorithm was fed with the peptidomics profiles of the BC-specific features (as identified in the training set), while the output of the modeling was evaluated on the test set. Optimization was performed via hyperparameter tuning, acting on the following parameters: number of trees, tree depth and number of features evaluated at each split (ranges specified in the Methods section). Highest performance in discriminating relapse from non-relapse patients was achieved using a set of 36 peptides (significant association at the significance level of 0.1, measured in at least six out of ten repeated analyses) (Supplementary Table S4). A detailed description of the 36 peptides is presented in Table 2. Following optimization, the prognostic 36-peptide model reached an accuracy of 100% (p-value < 0.001) in the training set. The optimal cut-off level of 0.47 for classification of relapse cases was determined in the training set based on Youden Index calculation.

**Table 2:** Characteristics of the 36 peptides selected for the prognostic model and hazard ratios measured in the entire cohort (n = 98).

Protein Name	Protein Symbol	Mass [Da]	CE time [min]	Hazard ratio
Collagen alpha-3(IV) chain	COL4A3	3349.54	30.97	HR: 8.75 (95% CI, 1.3–59.04), p = 0.026
—	—	4846.20	26.65	HR: 6.69 (95% CI, 1.95–22.99), p = 0.003
Peptidoglycan recognition protein 1	PGLYRP1	2187.99	27.08	HR: 4.86 (95% CI, 1.47–16.02), p = 0.009
Collagen alpha-1(I) chain	COL1A1	2488.11	27.95	HR: 4.26 (95% CI, 1.23–14.72), p = 0.022
Collagen alpha-1(I) chain	COL1A1	1522.68	22.23	HR: 3.84 (95% CI, 0.76–19.45), p = 0.104
Collagen alpha-1(I) chain	COL1A1	2103.96	33.08	HR: 3.74 (95% CI, 0.59–23.87), p = 0.163
Polymeric immunoglobulin receptor	PIGR	3556.62	23.96	HR: 3.64 (95% CI, 1.29–10.23), p = 0.014
Collagen alpha-4(IV) chain	COL4A4	2093.93	33.71	HR: 3.53 (95% CI, 1.3–9.56), p = 0.013
Collagen alpha-1(III) chain	COL3A1	2898.31	29.25	HR: 3.15 (95% CI, 0.6–16.58), p = 0.175
Collagen alpha-1(XIV) chain	COL14A1	3546.67	26.15	HR: 3.15 (95% CI, 0.99–10.02), p = 0.051
Forkhead box protein D2	FOXD2	3057.39	29.96	HR: 3.11 (95% CI, 1.18–8.16), p = 0.021
Collagen alpha-1(VI) chain	COL6A1	3136.39	24.55	HR: 2.65 (95% CI, 0.82–8.6), p = 0.105
Collagen alpha-1(III) chain	COL3A1	2564.15	23.00	HR: 2.4 (95% CI, 0.99–5.84), p = 0.054
Collagen alpha-1(V) chain	COL5A1	3385.59	25.54	HR: 2.37 (95% CI, 0.79–7.08), p = 0.123
Collagen alpha-1(III) chain	COL3A1	2323.05	22.39	HR: 2.34 (95% CI, 0.8–6.81), p = 0.12
Collagen alpha-1(V) chain	COL5A1	3722.78	21.94	HR: 2.32 (95% CI, 0.84–6.44), p = 0.106
Fibrinogen alpha chain	FGA	3314.48	20.21	HR: 2.22 (95% CI, 1.09–4.51), p = 0.028
—	—	9866.38	20.83	HR: 2.21 (95% CI, 1.04–4.68), p = 0.039
Collagen alpha-1(III) chain	COL3A1	2007.94	22.12	HR: 2.15 (95% CI, 0.84–5.48), p = 0.11
Ankyrin repeat domain-containing protein 36C	ANKRD36C	5574.25	23.16	HR: 2.12 (95% CI, 0.85–5.27), p = 0.105
—	—	8175.89	19.47	HR: 1.88 (95% CI, 0.75–4.71), p = 0.176
Collagen alpha-1(I) chain	COL1A1	2030.92	32.65	HR: 1.79 (95% CI, 0.44–7.2), p = 0.413
Collagen alpha-1(I) chain	COL1A1	2236.98	27.14	HR: 1.6 (95% CI, 0.66–3.91), p = 0.298
Collagen alpha-1(VIII) chain	COL8A1	3292.54	39.27	HR: 0.89 (95% CI, 0.37–2.16), p = 0.8
Nebulin	NEB	1135.49	27.79	HR: 0.84 (95% CI, 0.34–2.08), p = 0.714
Collagen alpha-1(I) chain	COL1A1	2170.97	27.53	HR: 0.76 (95% CI, 0.32–1.8), p = 0.533
Collagen alpha-1(I) chain	COL1A1	2319.04	33.85	HR: 0.64 (95% CI, 0.27–1.52), p = 0.31
Collagen alpha-2(IV) chain	COL4A2	2264.94	43.13	HR: 0.62 (95% CI, 0.37–1.03), p = 0.063
Collagen alpha-1(XI) chain	COL11A1	4169.93	33.60	HR: 0.56 (95% CI, 0.28–1.11), p = 0.096
Collagen alpha-1(I) chain	COL1A1	3432.59	31.95	HR: 0.53 (95% CI, 0.21–1.33), p = 0.179
Collagen alpha-1(XV) chain	COL15A1	1942.83	31.05	HR: 0.42 (95% CI, 0.11–1.55), p = 0.193
Collagen alpha-1(III) chain	COL3A1	1834.84	24.21	HR: 0.37 (95% CI, 0.15–0.91), p = 0.03
Collagen alpha-1(II) chain	COL2A1	1179.51	27.78	HR: 0.36 (95% CI, 0.12–1.09), p = 0.07
CD99 antigen	CD99	1954.97	25.45	HR: 0.27 (95% CI, 0.1–0.71), p = 0.008
Collagen alpha-1(I) chain	COL1A1	1795.79	24.93	HR: 0.26 (95% CI, 0.08–0.88), p = 0.03
Collagen alpha-1(III) chain	COL3A1	1396.62	26.63	HR: 0.19 (95% CI, 0.03–1.27), p = 0.088

### CE-MS based validation scores indicate an increased risk for relapse

**Figure 3:**
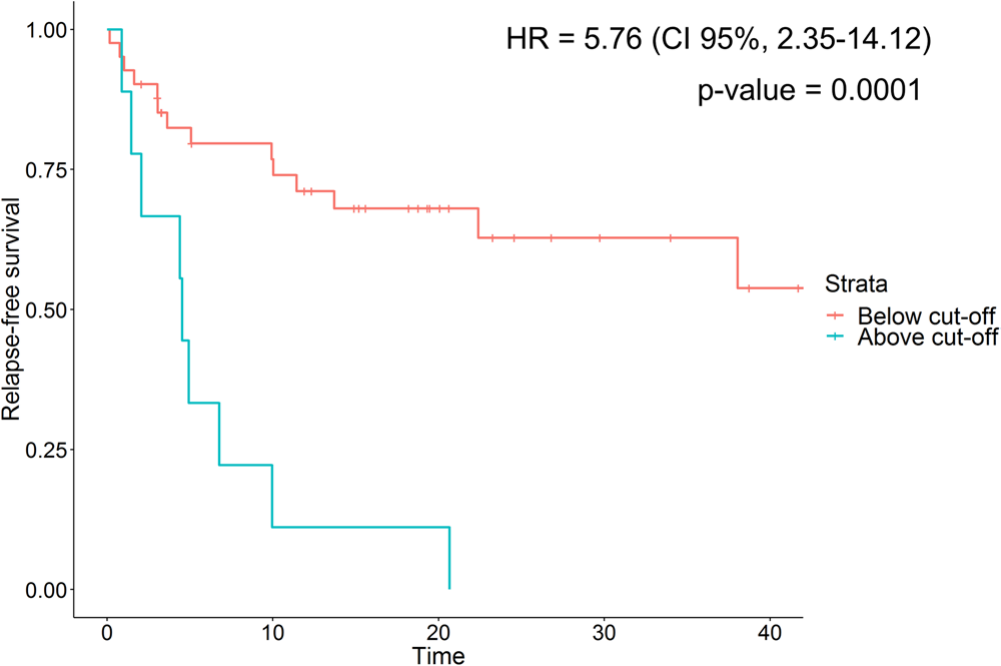
Performance of the Random Forest model predicting bladder cancer relapse (measured in the test set). Strata: red line – negative for relapse, blue line – positive for relapse, based on established cut-off value (0.47). *Abbreviations: HR* = *hazard ratio; CI* = *confidence interval*.

The prognostic value of the 36-peptide model was assessed in the independent test set. A significant prognostic potential was suggested based on Cox regression analysis (Table 3, Fig. 3; hazard ratio (HR) of 5.76 (CI 95%, 2.35–14.12), p-value = 0.0001). The developed model demonstrated high positive and negative predictive values, PPV = 66% and NPV = 100%. Calculated Harrell c-statistic indicated a fair predictive model capacity with a concordance of 0.64, while no additional variables have been found as confounding factors (Table 3).

**Table 3:** Univariate Cox regression analysis of potential predictor variables measured in the entire patient cohort and the developed machine learning model based on the test set. Abbreviations: CI = confidence interval.

Variable	Coefficient	CI l.95	CI u.95	p-value	Harrell c-statistic
Age	1.75	0.74	4.15	0.20	0.54
Gender (male)	1.02	0.99	1.04	0.22	0.57
Multiplicity (solitary)	0.87	0.47	1.62	0.66	0.54
**Stage**					
Tis	0.33	0.03	3.25	0.34	0.56
Ta	0.52	0.16	1.71	0.28	
T1	0.53	0.12	2.22	0.38	
T2	1.98	0.19	19.99	0.56	
T3	5.11e-8	0.00	Inf	0.99	
Tx	1.41	0.12	1.38	0.09	
**Grade**					
G2	1.38	0.66	2.89	0.39	0.56
G3	0.96	0.36	2.52	0.93	
Gx	4.53e-8	0.00	Inf	0.99	
36-peptide Model	5.76	2.35	14.12	0.0001	0.64

Among the 36 peptides that were included in the machine learning algorithm, three peptides could not be sequenced by using tandem mass spectrometry analysis (Table 2, Supplementary Table S4). This is most likely attributed to the rather large size and low abundance. The remaining 33 sequenced peptides included 23 various collagen alpha-1 fragments and single peptides from collagen alpha-2 (IV), collagen alpha-3 (IV) chain and collagen alpha-4 (IV). Additional sequenced peptides originated from fibrinogen (FGA), polymeric immunoglobulin receptor (PIGR), nebulin (NEB), peptidoglycan recognition protein 1 (PGLYRP1), forkhead box protein D2 (FOXD2), CD99 antigen (CD99) and ankyrin repeat domain-containing protein 36 C (ANKRD36C). Peptide characteristics along with the hazard ratios measured for each of the model building peptides based on all peptidomics datasets (n = 98) are reported in Table 2.

### Integration of the reported biomarkers in the context of BC pathology

To ascertain the validity of the reported urinary peptide biomarkers, a comparative analysis was performed considering available tissue proteomics datasets from previous studies of Latosinska *et al*.^21^ and Chen *et al*.^22^ involving tissue proteomics analysis in specimens derived from BC patients. In the study of Latosinska *et al*.^21^ tissue proteomics datasets from BC patients (n = 5 NMBC of Stage Ta and n = 6 MIBC of Stages T2+) were assessed, while in the study of Chen *et al*.^22^, tissue proteomics datasets from paired analysis of 4 BC patients (normal epithelium and cancerous lesions; Stages T1-T4) were evaluated. As described above, out of the 36 significant peptide biomarkers, sequences were annotated for 33 peptides, which corresponded to 19 distinct proteins. Among the 19 differentially excreted proteins, tissue expression was validated for 9 proteins via mass spectrometry proteomics. Those included collagen alpha-1 chains I, II, III, V, VI, XIV, XV, collagen alpha-2 chain IV and FGA (Supplementary Tables S5 and S6). In particular, decreased abundance of urinary collagen alpha-chain II (HR = 0.36) in BC relapse cases was in line with decreased tissue abundance as cancer stage progressed (Fold T2+/Ta = 0.25). Additionally, an increased abundance of urinary collagen alpha-1 chains V (HR = 2.33), VI (HR = 2.65) and FGA (HR = 2.22) was consistent with increase tissue abundance, along with cancer progression (Fold T2+/Ta of 1.47, 1.78 and 1.87 respectively).

In addition, BC gene expression data from the TCGA studies in tissue specimens from BC was assessed^23^, encompassing transcriptomics profiles from 406 patients (107 females and 299 males). Out of the 19 reported proteins (corresponding to 33 peptide sequence biomarkers, as reported in this study), gene expression was validated at the tissue level for all 19 proteins apart from FGA, where gene expression data was not available. According to this, unfavorable prognosis for BC relapse (i.e. higher relative expression levels correlating with increased risk for relapse) of Ankyrin repeat domain-containing protein 36 C (ANKRD36C; HR = 2.12), Forkhead box protein D2 (FOXD2; HR = 3.11) collagen alpha-1 chains I (HR = 1.94), III (HR = 1.77), IV (HR = 2.33), VI (HR = 2.65), XIV (HR = 3.15), collagen alpha-3 chain IV (HR = 8.75) and collagen alpha-4 chain IV (HR = 3.53) is in line with lower 5-year overall survival based on the TCGA gene expression data. In addition, biomarker nebulin (decreased in urine, correlating with increased risk for relapse; HR = 0.84) was also identified as a promising marker for BC based on the tissue gene expression data correlating with decreased overall survival (p = 0.041).

## Discussion

In this study, following-up on the previously published diagnostic markers based on CE-MS proteomics analysis^20^ and by enriching the analysis with longitudinal data, the prognostic performance of the CE-MS diagnostic panel was assessed for the risk of BC relapse. Notably, the previously published diagnostic panel for BC recurrence in a form of an SVM classifier (BC-106) was developed for the detecion of BC, not for prognosis of recurrence. However, it exhibited a significant, yet moderate prognostic value for BC relapse prediction. These initial results indicated a considerable prognostic value contained in at least some urinary peptides. When investigating the CE-MS derived profiling data at the single peptide level, several peptides were found significantly associated with a risk of BC relapse. Repeated statistical analysis using Cox regression was followed to shortlist the most valid features and integrate them into a machine learning model.

Mass spectrometry has been already applied for acquiring BC specific proteomics and metabolomics profiling data and several prognostic markers based on proteomics and metabolomics studies have been reported in the literature, highlighting the value of -omics features in improving BC management. Urine and serum proteomic-based biomarkers, like SPARC^24^, SH3 domain binding glutamic acid-rich protein like 3 (SH3BGRL3)^25^ have been recently reported as prognostic markers for BC. Moreover, according to recent metabolomics studies, i.e. in a first study investigating metabolic profiles of smokers and non-smokers with BC outcome^26^, catechol-O-methyltransferase (COMT), iodotyrosine deiodinase (IYD), tubulin tyrosine ligase (TTL) were correlated with BC survival, while in a study investigating population-based metabolic differences associated with BC^27^, high expression of lysine demethylase 2A (KDM2A) and prolyl 3‐hydroxylase 2 (P3H2) and low expression of mitochondrial malic enzyme 3 (ME3) was correlated with poor survival of African American BC patients.

In the present study, increase in the number of markers (peptides) in high-dimensional classifier resulted in improved performance, which is in good agreement with previous studies showing a clear advantage of using multiple features as compared to single markers for predictive disease modeling^28–30^. This observation seems consistent, provided that the variables are truly associated with the investigated outcome to positively influence the model training phase. High performance of the established model indicated that the 36-peptide model is sufficient to embrace the heterogeneity of BC patients and forecast an accurate prognosis.

From the 36 peptides, corresponding to 19 distinct proteins, we were able to obtain sequence information for 33 of them. The majority of sequenced peptides originated from multiple collagen fragments (mainly collagen alpha-1) and were found associated with both, good and poor prognosis depending on a specific sequence. Based on the literature and our previous CE-MS studies, collagen increase and decrease are both involved in tumor progression^20,31^, as collagen initially acts as a barrier and collagenases, such as metalloproteinases (MMPs), which degrade it to expose active sites and promote a pro-tumorigenic microenvironment to facilitate tumor progression. Collagen cross-linking and thickening is then necessary during extracellular matrix (ECM) remodeling and invasion. Elevated levels of urinary fibrinogen have already been reported in BC patients and associated with tumor invasiveness^32,33^. As such, presence of the FGA among the peptides with high prognostic value is further confirming its association with the disease. Increased levels of PIGR were also found associated with a higher risk of relapse (Table 2). PIGR is a member of the immunoglobulin superfamily, involved in transcytosis of IgA and other immune complexes. Although it was found in the tumor tissues of BC patients, no correlation with tumor stage or grade could be established^34^. Nevertheless, association with disease relapse was, to our knowledge, not studied. Another immune-related protein associated with higher chance of BC relapse was peptidoglycan recognition protein 1 (PGLYRP1). Interestingly, its role in anti-cancer defense was recently suggested via formation of cytotoxic complexes with heat shock protein 70^35^. Transcription factor forkhead box protein D2 (FOXD2) was found to be related with poor prognosis. Additionally, nuclear FOXOs are known to mediate cell cycle arrest and promote apoptosis^36^. With regards to BC, a recent analysis of long non-coding RNAs linked high FOXD2-AS1 expression to BC progression and recurrence by acting on Act/E2F1 axis^37^. Among the peptides indicative of good prognosis and lower risk of BC relapse was CD99 antigen (CD99), which in line with the reports suggesting it’s oncosuppressive role in BC^38,39^.

Validation of the reported peptide biomarkers at the tissue level was possible through a comparative analysis with available mass spectrometry acquired tissue proteomics datasets^21,22^. The comparative analysis confirmed the decrease in protein abundance of collagen alpha-chain II in BC relapse cases and increased urinary abundance of collagen alpha-1 chains V, VI and FGA. Importantly, for collagen alpha-1 chain V, additional reports on immunohistochemical staining (i.e increased staining in MIBC) support the validity for increased tissue/urine abundance in advanced BC^40^. Yet, the consistency between the tissue protein abundance and urine excretion has to be considered with caution, as in several observations, the differential abundance at the tissue level was not significant (at the level of comparison between 5 NMIBC and 6 MIBC BC proteomics datasets, considering 60% frequency threshold). In parallel, tissue data from gene expression analysis in BC tissue specimens (TCGA)^23^ confirms ANKRD36C, FOXD2, collagen alpha-1 chains I, III, IV, VI, XIV, collagen alpha-3 chain IV and collagen alpha-4 chain IV as unfavorable and nebulin as favorable prognostic markers for BC outcome.

Given the high heterogeneity of BC, the results of the study are promising. Among the possible applications, use of the model as a tool for patient stratification e.g. for clinical trials is anticipated, as it would enable enriching for patients that are highly possible to develop a relapse. These patients may consequently possibly benefit from appropriate preventive therapeutic intervention.

Due to missing clinical parameters, a direct comparison of the performance of the prognostic model that was developed in this study with other methods to predict relapse was not possible. However, we were able to compare the predictive value with published reports on the most prominent risk calculators. In the report by Xylinas *et al*., the authors evaluated the performance of both, EORTC risk tables and the CUETO scoring model using the retrospective cohort consisting of 4689 patients with NMIBC^16^. Calculated concordance indexes of the models for recurrence and progression prognosis were 0.597 and 0.662 for EORTC, and 0.523 and 0.616 for CUETO model, respectively. Comparison with the original estimates published by EORTC and CUETO indicated reduced discriminative ability of the model in the reported validation study. As such, the authors pointed out the poor discrimination ability of the scoring models for both disease recurrence and progression in NMIBC patients, stressing the need for improvement of tools for risk prognosis^16^. Given that the model developed in our study reached the concordance of 0.639, it represents a good alternative to currently used prognosticators, introducing a truly personalized approach to relapse prognosis, based solely on urinary peptide profiles of BC patients.

Several limitations are present in this study. The low number of samples has an obvious influence on the significance of model-building features and the model itself. Moreover, the performance of the final model was assessed on the test set originating from the same cohort, which might introduce a bias in the evaluation. An independent validation cohort would be of added value in confirming the predictive capabilities of the developed approach. Expansion of the dataset to include more BC patients may improve the performance, providing the learning algorithm with more examples of the molecularly variable cases. Moreover, lack of certain parameters in the clinical data made it impossible to compare the developed model to standard-of-care risk calculators. Overall, these promising preliminary results on the development of machine learning model based on peptidomics signatures for the prediction of BC relapse highlight the potential of proteomics technology in clinical applications. Efforts to enrich the patient database and further improve the model are foreseen in the future.

To sum up, risk stratification strategies are essential for more personalized management of BC. Prediction of BC relapse can assist in guiding intervention and build the foreground for prediction of treatment response. Incorporation of the presented model in clinical trials to further establish its clinical use and potential impact on decision-making will be pursued.

## Methods

### Patient population

For this study, previously acquired and published CE-MS profiling data^20^ were selected to be further analyzed prospectively. Peptidomics profiling datasets from patients initially recruited at Erasmus MC, Rotterdam, with available follow-up information were included in this study. This resulted in the inclusion of 98 BC patients. Sample and data collection was performed in accordance with local ethics requirements and the Declaration of Helsinki. Written informed consent was obtained from all participants prior to study enrollment and approved by Ethics Committee. For this meta-analysis (follow-up investigation), ethics approval was obtained by the Ethics Committee of Hannover Medical School (MHH), under the identifier Nr. 3274–2016. As previously described^20^, all urine samples were collected prior to cystoscopy and the patients were followed-up for a period of up to 5 years. The presence of BC was considered based on the cystoscopy results, while tumor stage was defined according to the TNM (tumor nodes metastases) classification^41^, following histological examination of tissue specimens during the biopsy. Event endpoints (relapse/non-relapse) were assigned according to the following criteria: (a) the timepoint when the urine measurement was acquired was considered as baseline, (b) the timepoint of the first relapse event was considered to define the survival time for bladder cancer patients (Event = 1) and (c) relapse-free patients were considered as non-event for controls (Event = 0). The cohort characteristics are summarized in Table 1 and the full list of patient clinical data is given in Supplementary Table S1.

### Processing of urine for peptidomics analysis and data analysis

The peptidomics datasets that were analyzed in this study, were originally acquired in the context of the study by Frantzi *et al*.^20^. The methodologies for urine sample processing and collection of peptidomics data were described in detail in the respective publications^42,43^. CE-MS analysis was performed using a P/ACE MDQ capillary electrophoresis system (Beckman Coulter, Fullerton, USA) on-line coupled to a MicroTOF MS (BrukerDaltonic, Bremen, Germany), as described previously^20^. CE-MS data was analyzed with MosaiquesVisu internal software^43,44^. Normalization of the CE-MS data was performed based on 29 collagen fragments that serve as internal standards, as previously described^45^. The obtained spectra were analyzed with Proteome Discoverer 1.2 (Thermo Scientific) (precursor mass tolerance of 5 ppm and fragment mass tolerance of 0.05 Da) and searched against UniProt human non-redundant database. Oxidation of methionine and proline were considered as variable modifications^46^. Detected peptides were annotated, matched and deposited in a Microsoft SQL database (Human Urinary Proteome Database^47,48^) and used as an input in the present study.

### Statistical analysis

Based on the available follow-up information, patients experiencing recurrence or progression were considered as relapse cases (Event = 1), while patients without relapse were classified as controls (Event = 0). Only peptides detected in at least 30% of all samples were considered for the analysis (k = 1046). Scaling of peptide abundance values (log10 transformation) was performed in the pre-processing step. Additionally, missing values, often present in the peptidomics datasets due to biological (selective expression in pathological or physiological process) and/or technical factors (abundance below the limit of detection), were replaced by zeros. The prognostic performance of the previously published peptide panel for diagnosis of BC recurrence (BC-106) was assessed through Cox regression. Association of peptide abundance with relapse event was assessed in the training set (n = 48) using Cox regression analysis. The analysis was repeated ten times on 70% of randomly selected samples. For each peptide, a number of permutations in which it was found significant were calculated (significance level of 0.1) and feature sets consisting of peptides appearing significant in all ten (10/10), nine (9/10), eight (8/10), seven (7/10) and six (6/10) repeated analysis were created. Subsequently, machine learning prognostic models were developed based on these significant peptide sets. Statistical analysis was performed using R statistical software version 3.3.3.

### Machine learning model development

A machine learning algorithm was implemented using package “H2O” in R statistical software. H2O (www.h2o.ai) is an open-source machine learning platform allowing implementation of many supervised and unsupervised machine learning algorithms^49^. The machine learning algorithm (script) is described in the Supplementary Script. Three-fold cross-validation was applied in the model building step. Models based on the Random Forest algorithm including different sets of biomarkers (from Cox regression analysis, as described above) were trained separately and optimized to identify the best performing set of biomarkers. The parameters selected for the optimization (ranges specified in brackets) included: a) number of trees (ntrees) [5,50], b) maximum tree depth (max_depth) [2,10], and c) number of active, randomly picked predictor columns for the dataset (mtries) [−1,20]. The optimization process involved acting on these parameters by testing how different values contribute to overall model performance in the independent validation set. The optimal parameters selected for the final model were as follows: ntrees = 11, max_depth = 3, mtries = default (−1).The optimal cut-off value was estimated in the training set, based on the the Youden index. The accuracy was further calculated by comparison of predicted classes to training set labels (Confusion Matrix), as Accuracy =$\frac{(Sensitivity+Specificity)}{2}$. The prognostic performance of the model was assessed based on the Cox regression analysis. Positive predictive value (PPV), negative predictive value (NPV) and concordance (Harrell C-statistic) were calculated to assess the goodness of fit of the model.

## Supplementary information

Supplementary Data

Supplementary Script

## Data Availability

The analyzed datasets generated during the current study can be available from the corresponding author on reasonable request.
