# Supplementary material for: Urinary peptide panel for prognostic assessment of bladder cancer relapse
Source: Sci Rep. 2019 May 21;9:7635. doi: 10.1038/s41598-019-44129-y (PMC6529475; doi:10.1038/s41598-019-44129-y)
Supplement: Supplementary file 2 — Supplementary Script [file 41598_2019_44129_MOESM2_ESM.docx]

**Urinary peptide panel for prognostic assessment of bladder cancer relapse**

Magdalena Krochmal^1^, Kim E. M. van Kessel^2,3^**,** Ellen C. Zwarthoff^2^, Iwona Belczacka^1^, Martin Pejchinovski^1^, Antonia Vlahou^4^, Harald Mischak^1^, Maria Frantzi^1*^

^1^Mosaiques Diagnostics GmbH, Hannover, Germany;

^2^Department of Pathology, Erasmus MC Cancer Institute, Erasmus Medical Center, Rotterdam, the Netherlands;

^3^Department of Urology, Erasmus MC Cancer Institute, Erasmus Medical Center, Rotterdam, the Netherlands.

^4^Biotechnology Division, Biomedical Research Foundation, Academy of Athens (BRFAA), Athens, Greece.

**Supplementary Data**

**R script for generation of the Random Forest Model**

#load required libraries

library(rpart)

library(xlsx)

library(caret)

library(h2o)

library(pROC)

library(rms)

h2o.init()

### Specify the working directory with the input files

setwd("C:/R scripts/Random Forest model/")

## load file with list of peptides (after feature selection) to be selected from the whole data matrix

peptides <- read.xlsx("Input_peptide_list.xlsx", sheetIndex = 1)

pep_list <- colnames(peptides)

############### set model parameters

## number of trees

trees <- c(20)

result_rf <- data.frame()

result_all <- data.frame()

for (j in 1:length(trees)) {

print(j)

for (i in 1:ncol(peptides)) {

print(i)

set <- pep_list[i]

pept <- as.vector(peptides[,i])

# load clinical data for training and test sets

training_set <- read.xlsx("Input_training_set_clinical.xlsx", sheetIndex = 1)

rownames(training_set) <- training_set$Auswert_ID

test_set <- read.xlsx("Input_test_set_clinical.xlsx", sheetIndex = 1)

rownames(test_set) <- test_set$Auswert_ID

# load training data matrix

training_mat <- read.table("Input_training_matrix.txt", header = T, sep = "\t")

rnt <- training_mat$fidAuswertung

training_mat <- as.data.frame(training_mat[,colnames(training_mat) %in% pept])

training_mat <- apply(training_mat, 2, log10)

rownames(training_mat) <- rnt

training_mat <- merge(x = training_mat, y = training_set[,c(3,9)], by = "row.names", all.x = T)

row.names(training_mat) <- training_mat$Row.names

training_mat <- training_mat[,-c(1)]

training_mat <- subset(training_mat, select=-c(Auswert_ID))

training_mat[is.na(training_mat)] <- 0

# load test data matrix

test_mat <- read.table("Input_test_matrix.txt", header = T, sep = "\t")

rnts <- test_mat$fidAuswertung

test_mat <- test_mat[,colnames(test_mat) %in% pept]

test_mat <- apply(test_mat, 2, log10)

rownames(test_mat) <- rnts

test_mat <- merge(x = test_mat, y = test_set[,c(3,9)], by = "row.names", all.x = T)

row.names(test_mat) <- test_mat$Row.names

test_mat <- test_mat[,-c(1)]

test_mat <- subset(test_mat, select=-c(Auswert_ID))

test_mat[is.na(test_mat)] <- 0

########### random forest

training_mat$Event <- as.factor(training_mat$Event)

fit_rf <- h2o.randomForest(y = "Event",seed = 11,

training_frame =as.h2o(training_mat),

nfolds = 3, ### parameter for tuning

ntrees = trees[j]

,balance_classes = TRUE ### parameter for tuning

,mtries = 4 ### parameter for tuning

,max_depth = 11 ### parameter for tuning

)

# save the model

dir.create(paste("Output_Random_Forest_model_", trees[j],"_trees_",set, sep=""))

new_dir <- paste("Output_Random_Forest_model_", trees[j],"_trees_",set,sep="")

model_path <- h2o.saveModel(object=fit_rf, path=new_dir, force=TRUE)

### To load ther model from disc (example)

#model <- h2o.loadModel(path = "./Output_Random_Forest_model_20_trees_selected_peptides/DRF_model_R_1533648743237_2")

pred_rf <- as.data.frame(predict(fit_rf, as.h2o(training_mat)))

rownames(pred_rf) <- rownames(training_mat)

### calculate Youden Index for cut-off

rocobj <- roc(training_mat$Event, pred_rf[,2])

c2 <- coords(rocobj, x="best", input="threshold", best.method="youden")

### Performence in training set (Cox regression)

train_cox <- merge(x = pred_rf, y = training_set[,c(3,9,10)], by = "row.names", all.x = T)

train_cox$Event <- as.factor(train_cox$Event)

train_cox$class <- ifelse(train_cox$p0>c2[1],0,1)

train_cox$class <- as.factor(train_cox$class)

cf_tr <- confusionMatrix(train_cox$class, train_cox$Event)

### Performence in test set (Cox regression)

pred <- as.data.frame(predict(fit_rf, as.h2o(test_mat)))

rownames(pred) <- rownames(test_mat)

test_cox <- merge(x = pred, y = test_set, by = "row.names", all.x = T)

test_cox$Event <- as.factor(test_cox$Event)

test_cox$class <- ifelse(test_cox$p0>c2[1],0,1)

test_cox$class <- as.factor(test_cox$class)

cf_tst <- confusionMatrix(test_cox$class, test_cox$Event)

result_rf[i,"Peptide_Set"] <- set

result_rf[i,"# of Trees"] <- trees[j]

result_rf[i,"Set"] <- "training"

result_rf[i,"Accuracy"] <- cf_tr$overall["Accuracy"]

result_rf[i,"p_val_training"] <- cf_tr$overall["AccuracyPValue"]

result_rf[i,"Specificity"] <- c2[2]

result_rf[i,"Sensitivity"] <- c2[3]

result_rf[i,"Cut-off"] <- c2[1]

result_rf[i,"Set_test"] <- "test"

result_rf[i,"Accuracy_test"] <- cf_tst$overall["Accuracy"]

result_rf[i,"p_val"] <- cf_tst$overall["AccuracyPValue"]

result_rf[i,"Specificity_test"] <- cf_tst$byClass["Specificity"]

result_rf[i,"Sensitivity_test"] <- cf_tst$byClass["Sensitivity"]

cox_score_test <- coxph(formula = Surv(time = test_cox$Survival_time, event = as.numeric(test_cox$Event)) ~ test_cox$class,

data = test_cox)

sum <- summary(cox_score_test)

result_rf[i,"Cox_test coef"] <- sum$conf.int[1]

result_rf[i,"Cox_test coef l.95"] <- sum$conf.int[3]

result_rf[i,"Cox_test coef u.95"] <- sum$conf.int[4]

result_rf[i,"Cox_test p"] <- sum$coefficients[5]

print(cf_tst$overall["Accuracy"])

}

result_all <- rbind(result_all,result_rf)

}

### save results to a file

write.xlsx(result_all, "Output_Random_forest_performance_metrics.xlsx", row.names = F)
